# High Index of Suspicion Has Life-Changing Results in an Older Adult Patient

**DOI:** 10.1016/j.chpulm.2025.100223

**Published:** 2025-10-13

**Authors:** Dagan Adi, Onn Amir, Efrati Ori

**Affiliations:** a Pediatric Pulmonary Unit and the National Center for Cystic Fibrosis, Edmond and Lili Safra Children's Hospital, Tel-Hashomer, Israel; b Pulmonary Institute, Sheba Medical Center, Tel-Hashomer, Affiliated to Sackler Medical School, Tel Aviv University, Tel-Hashomer, Israel

## Abstract

A 70-year-old man with progressive bronchiectatic lung disease, cirrhosis, and hepatocellular carcinoma supposedly secondary to hepatitis B was referred to our institution for further evaluation. A detailed medical history identified the patient as of Romanian Jewish ethnicity with no prior familial or genetic disease reported. As a young child, he reported hospitalization for failure to thrive but no respiratory complaints. In adulthood, he underwent a cholecystectomy for gallbladder stones and successful fertility treatment resulting in 2 children. His liver and lung diseases became clinically significant in his late 40s. Chronic productive cough and repeated episodes of hemoptysis led to a diagnosis of bilateral bronchiectasis, particularly affecting the upper and middle lobes. A few years prior, he underwent angiography and embolization of a tortuous left bronchial artery due to massive hemoptysis. The patient reported anorexia and significant weight loss of 20 kg, accompanied by intermittent diarrhea, which were attributed to his underlying lung and liver conditions.

## Physical Examination Findings

On initial examination, the patient was afebrile with stable vital signs measuring heart rate of 78 beats/min, BP of 105/70 mm Hg, respiratory rate of 12 breaths/min, and oxygen saturation of 97% on room air. Examination revealed a cachectic, pale male appearing chronically ill, with multiple diffuse hematomas. He was alert and oriented, and no increased work of breathing was observed. Oropharynx was clear with no jugular venous distention. He had a barrel chest structure, and auscultation revealed low entry of air and bilateral diffuse crackles. His cardiac examination demonstrated regular rate and rhythm. The abdomen was distended with severe splenomegaly, but no ascites were palpated. There was no clubbing of his fingernails. The remainder of the physical examination was normal.

## Diagnostic Studies

Hemoglobin level was 10.9 g/dL, and WBC count was 4,500/μL, with lymphocytes of 400/μL and platelets of 29,000/μL. Sodium level was 130 mmol/L, potassium level was 4.6 mmol/L, blood urea nitrogen was 17 mg/dL, and creatinine was 0.89 mg/dL. Total bilirubin was 2.39 mg/dL, direct bilirubin was 1.49 mg/dL, alanine aminotransferase was 18 International Units/L, alkaline phosphatase was 281 International Units/L, albumin 3.8 was g/dL, glucose 106 was mg/dL, and international normalised ratio was 1.32. He had normal immunoglobulin levels and negative vasculitis workup. Sweat test was not completed due to lack of sweat. Stool elastase was 169 μg/g (normal > 200 μg/g). Spirometry demonstrated obstructive disease with FEV_1_ measured at 1.83 L (62% predicted), FVC at 2.92 L (76% predicted), and FEV_1_/FVC at 0.63 (normal > 0.8), confirming impaired lung function. Bronchoalveolar lavage cultures grew *Mycobacterium abscessus*, *Staphylococcus aureus*, and *Pseudomonas aeruginosa*.

### Imaging

**Figure 1:**
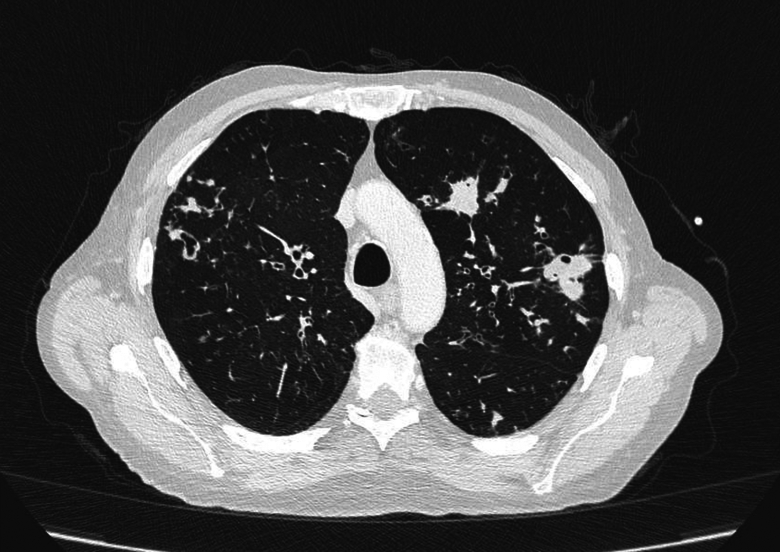
Chest CT scan demonstrates bilateral upper lobe bronchiectasis and a cavitation in the left upper lobe.

**Figure 2:**
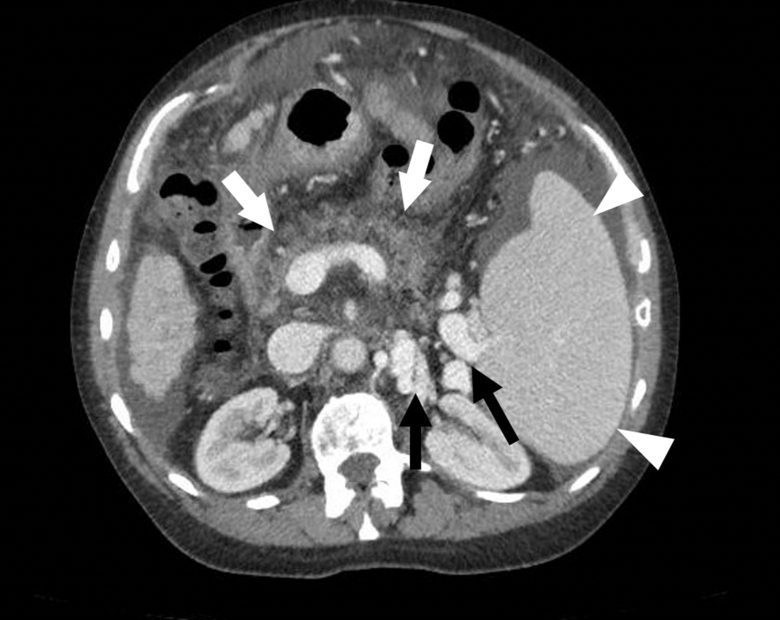
Abdominal CT scan highlights splenomegaly (white triangles), atrophy of the pancreas (white arrows), and varices secondary to portal hypertension (black arrows).

Representative imaging included a chest CT scan showing bilateral bronchiectasis prominently in the right upper lobe, right middle lobe, and left upper lobe, with mucous plugs and a cavitation in the left upper lobe (Fig 1). Additionally, an abdominal CT scan revealed a cirrhotic liver with splenomegaly and an atrophic pancreas (Fig 2).

*What is the diagnosis* *?*

*What study should be conducted next?*

*Diagnosis:* Cystic fibrosis

*Next steps:* Genetic sequencing of the cystic fibrosis transregulator gene to confirm the diagnosis

## Discussion

Cystic fibrosis (CF) is typically diagnosed in the first months of life, allowing most patients to benefit from long-term follow-up at specialized CF centers. However, a significant minority of individuals, approximately 20%, are diagnosed in adulthood, with only a small proportion diagnosed at > 60 years of age. This highlights the importance of considering CF as a potential diagnosis across the human lifespan. Adult patients presenting with unexplained bronchiectasis, chronic rhinosinusitis, recurrent pancreatitis, cholestatic liver disease, infertility, or hematologic abnormalities, such as those described in this case, should undergo testing for CF.

Diagnosing CF in adulthood presents unique challenges due to its variable presentation, the clinician’s familiarity with atypical forms of CF, and even the impact of environmental factors such as smoking or alcohol use on clinical manifestations. Once clinical suspicion is raised, confirmatory testing involves a sweat chloride assay, with values ≥ 60 mmol/L considered suggestive of CF, and genetic testing for 2 CF-causing mutations in trans. *CFTR* mutations can be categorized into 6 functional classes based on their impact on the CFTR protein. These classes range from defective protein production (class I) to impaired channel regulation and stability (class VI). Such classifications help explain phenotypic variability and guide personalized treatment approaches.

Among CF mutations, F508del is the most prevalent worldwide, occurring in approximately 80% of patients. As a class II mutation, it results in misfolding of the CFTR protein, complete loss of function, and severe disease phenotypes. Similarly, W1282X, a class I mutation, produces a truncated CFTR protein associated with a complete loss of function and severe disease. Although globally rare (approximately 1%), W1282X is notably prevalent in the Ashkenazi Jewish population, with a frequency of approximately 35%. In contrast, class IV mutations, such as D1152H, retain residual protein activity, leading to milder symptoms and a broader clinical spectrum. Compound heterozygotes carrying 1 mutation with residual function (D1152H) and 1 severe mutation (eg, F508del, W1282X) may have a relatively mild phenotype during childhood, often remaining pancreatic sufficient, yet develop worsening symptoms and progressive disease over time. Understanding the specific mutation types in each case becomes crucial for tailoring treatment, particularly with *CFTR* modulator therapies.

Specific therapies are available to address the symptoms and complications of CF. For instance, inhaled antibiotics, such as colistin, tobramycin, or aztreonam, play a vital role in managing chronic pulmonary infections, particularly those involving *P aeruginosa*. Recombinant dornase alfa (DNase) is used to improve airway clearance by reducing mucus viscosity. Although these therapies do not target the underlying defect in *CFTR* function, they remain essential in reducing morbidity, improving lung health, and enhancing overall quality of life in patients with CF.

Treatment with *CFTR* modulators has revolutionized the management of CF, offering targeted therapies that modify the underlying pathophysiology of the disease. *CFTR* modulators are small-molecule therapies that address specific classes of *CFTR* mutations, improving *CFTR* protein function. Elexacaftor-tezacaftor-ivacaftor (ETI), a triple combination therapy, has shown significant benefits for patients with responsive mutations, such as F508del and D1152H. ETI improves *CFTR* function by addressing protein misfolding and enhancing chloride channel activity. Clinical studies have demonstrated that ETI is associated with enhanced lung function (FEV_1_), reduced pulmonary exacerbation rates, weight gain, and improved quality of life. Beyond these benefits, emerging evidence suggests that ETI may have a role in managing CF-related complications, such as mycobacterial pulmonary disease, with several reports indicating higher rates of infection eradication in patients treated with this therapy. Research is ongoing to assess its broader applications and impacts on long-term outcomes.

In recent years, *CFTR* modulators such as ETI have also been introduced for select patients with CF after liver transplantation. However, concerns remain regarding potential drug interactions with immunosuppressive agents. A recent systematic review highlighted significant improvements in lung function for patients after liver transplant treated with ETI, with most patients tolerating the therapy well. Nevertheless, liver function abnormalities were reported in a subset of cases, underscoring the necessity for close monitoring of liver enzyme levels and immunosuppressive drug concentrations during therapy. Although ETI provides hope for improving outcomes in complex cases like these, further studies are needed to evaluate its safety and efficacy in diverse CF populations.

### Clinical Course

*CFTR* gene sequencing results found compound heterozygote pathologic mutations, W1282X and D1152H, and diagnosis of CF was determined.

Before genetic diagnosis, the patient began aggressive therapy including daily respiratory physiotherapy and inhaled hypertonic saline along with a combination of inhaled and systemic antibiotics. After stabilization of lung disease, he underwent successful liver transplantation. Pathologic studies demonstrated severe biliary cirrhosis and cholestatic disease and a noninvasive 2-cm-sized tumor.

After a final diagnosis of CF was established, we added specific treatments including ETI modulator therapy, inhaled DNAse, pancreatic enzyme, and fat-soluble vitamin replacement. These specific treatments resulted in significant clinical improvement including rapid weight gain and cessation of chronic abdominal pain and diarrhea, decline in cough and sputum production with negative mycobacterial sputum culture, a rise in lung function to FEV_1_ 86% predicted, and normal liver function tests.

## Clinical Pearls

1. *A constellation of bronchiectasis with* P aeruginosa *and* M abscessus *in sputum, cholestatic liver disease, pancreatic insufficiency, and infertility should raise suspicion for diagnosing CF. A comprehensive history and physical examination are essential to focus on all systems involved and make a connection between them.*
2. *Diagnosis of CF, even in late adulthood, enables access to CF-specific therapies, such as inhaled DNase, inhaled antibiotics, and CFTR modulators, significantly improving patient outcomes and quality of life.*
3. *CFTR modulator therapies significantly improve pulmonary and gastrointestinal outcomes in patients with CF. Gene sequencing is critical for identifying those eligible for this targeted treatment.*

## Financial/Nonfinancial Disclosures

None declared.
